# Clifton

**Published:** 1889-06

**Authors:** John Beddoe


### CLIFTON.

BY

JOHN BEDDOE, M.D., F.R.S., &c.

CLIFTON has grown in little more than a hundred years from a village, which strove to combine rustic simplicity with the quality of a fashionable watering-place, into the extensive and populous West-end of the neighbouring city. The Clifton of to-day occupies nearly a square mile of surface, bounded on the east and north-east by Bristol and its northern suburbs; on the south by the floating harbour of Bristol; on the south-west by the Avon in its deep and picturesque gorge; and on the west and north-west by the same, but with the interposition of those high and breezy limestone plateaux, covered with short sweet grass and dotted with hawthorn bushes, all blushing and fragrant in the month of May, which are called Clifton and Durdham Downs.

Along the harbour stretches a narrow alluvial strip, which, with the lower portion of the adjacent slopes, is occupied by the district called the Hotwells, densely peopled by a miscellaneous but largely sea-faring community, many of whom dwell in old mansions of some pretension, now cut up into tenement houses.

The higher slopes, and the rolling ground abutting on the downs, are much more thinly inhabited; the houses are of modern erection, generally of oolitic freestone or of the native limestone, interspersed with open spaces and garden ground. This district is occupied by those who have resorted to Clifton for social, educational, or sanitary reasons, and by the upper class of Bristolians, with those who minister to their service and purveyance; and here are the principal hotels and lodging-houses, the College, the Assembly Rooms, the Academy of Arts, &c. The best portion of this district consists, like the adjacent downs, of carboniferous limestone, unfortunately capped in places with a thin layer of boulder clay: lower down are found the millstone grit, ferruginous marl, and beds belonging to the new red sandstone. The surface lends itself well to drainage, both superficial and deep; the roads dry easily, and water-carts are welcome in the spring.

Builders are commonly said to have spoiled, at Clifton, one of the finest sites in Europe. They could not spoil it altogether, nevertheless. The crescents, towering one above the other, have a stately aspect from the valley to the south, as one rushes along the railway towards Exeter. The Suspension Bridge, though far from being, as it once was, unrivalled in span throughout Europe, is still a marvel of beauty and a triumph of engineering: one must go as far as to America to find its superior in loftiness and in picturesqueness of surroundings. It affords access, moreover, to the Leigh Woods, a tract of very beautiful sylvan scenery, the delight of landscape painters, such as is nowhere else to be found in the immediate neighbourhood of a great city.

The gorge through which the Avon cuts its deep and devious way is still magnificent, though some of its salient angles have been rubbed away in the interest of commerce, and, as Robert Hall said, "the Corporation of Bristol have sold the sublime and beautiful for sixpence the cart-load." At the bottom, within sight of the bridge, is the once famous Bristol Hotwell. The blasting and quarrying operations just alluded to have cleared away the point whence it used to spring; but the well has been recovered, and the water of the river successfully excluded from it.

It was this spring which first made Clifton a place of resort for invalids. In the latter part of the last century dyspeptics, diabetics, and sufferers from gravel and stone, resorted to it with hope and confidence, and *poitrinaires* from the north and east came to try the virtues of the water as well as of the mild climate. Soft, relaxing air was not then out of favour, as it is now-a-days; and the warm undercliff of Dowry Square was the favourite residence. There lived and flourished Dr. Thomas Beddoes, that eccentric genius, one of the fathers of chemistry, the master of Humphrey Davy, and the inventor of inhalation.

The water is not, as many not unnaturally suppose, a cooler dilution of that of Bath, bearing to the latter the relation

"Of moonlight unto sunlight, and of water unto wine."

It is cooler, indeed (about 78° Fahr.),* and much less mineralized, but its constituents are very differently proportioned. That of Bath is a lime-sulphate water, essentially, whereas in ours the chief constituent, the carbonate of lime, preponderates over the sulphate. The

* Weber says 74°; W. W. Stoddart, 70°; Macpherson, 74°.

former water is more suitable for baths, the latter for drinking.

It contains per gallon, according to Herapath:

| | | |
|---|---|---|
| Carbonate of Lime | 17.70 | grains. |
| Sulphate of Lime | 9.87 | ,, |
| Chloride of Sodium | 5.89 | ,, |
| Sulphate of Soda | 3.01 | ,, |
| Chloride of Magnesium | 2.18 | ,, |
| Nitrate of Magnesia | 2.91 | ,, |

with smaller quantities of carbonate of magnesia and of iron, of bitumen and of silica, and some impregnation of carbonic acid and of nitrogen gas.* These proportions are intermediate between those of Buxton and those of Bath; but the resemblance to Buxton is the nearer. The water also stands between Contrexéville and Buxton in most respects, but is rather more mineralized than either of these.

Another near analogue appears to me to be the Stadtbrunnen of Wildungen, which, however, is cold and comparatively concentrated. Now the Wildungen waters, like those of Coutrexéville, have "a special and well-deserved reputation for their curative powers in gravel and the lithic-acid diathesis." † Again, the Clifton water had at one time a great reputation in diabetes. This we now regard as having been imaginary or fictitious; but it may be worth while to observe that the Bethesda water

* Dr. Griffin, who has studied the water within the last few years, and made some careful analyses, has established the existence of copper therein, but has been unable to detect boracic acid. He has found the water susceptible of preservation in good condition for an indefinite time: this quality, among others, doubtless led to its having been supplied, in former years, as an ordinary table-water to the royal family. Mr. F. W. Stoddart, who has also analysed the water, found the same aggregate amount of solids (4.4 per 1000) as Herapath did, but with slight differences in proportion—rather more sulphuric acid, and less of nitric acid and chlorine; rather less lime, and more magnesia and silica.

† Althaus and Braun.

of Wisconsin, which a few years ago acquired an empirical reputation in diabetic cases, is an earthy water with about the same proportion of its leading constituent (bicarbonate of lime) as Clifton water has. Of its merits as a beverage in many cases of dyspepsia the practitioners of Clifton have no doubt; and on the whole it is much to be regretted than a recent attempt to bring the water to the upper or fashionable level of the town, and to establish a Spa there, has broken down from lack of funds. The observatory on the summit of Clifton Down, with its magnificent prospect, would afford an almost unrivalled site for a pumproom.

The gradual desertion of the Hotwells was coincident with, and probably mainly due to, the extension of building over the upper levels of Clifton, and the transference thither of the wealthy and fashionable elements of society. *Poitrinaires* and rheumatic patients continued to resort hither; but instead of the somewhat muggy district at the bottom of the hill, they now sought the sunniest and best aspects on the top of it, such as Sion Hill, Prince's Buildings, the York Crescent, and The Mall. Scotch invalids were very numerous at this period, as is still testified by the names of Caledonia and Melrose Place, Hope Chapel Hill, &c.; and doubtless the difference of climate between most parts of Scotland and the West of England is sufficiently sensible to justify, in many cases, considerable expectations as to the result of migration from the one to the other.

The present generation, however, has witnessed a further change, which may be summed up thus. Clifton has ceased to be a watering-place: it is hardly even an invalid-resort: it is the West-end of Bristol, a large educational and social centre, and a place of sojourn for tourists on account of its local advantages and its beautiful environs. Even the climate has changed: the average annual depth of rain has increased from about 30 to 34 inches; while the atmosphere, always dry compared with that of most parts of Britain, has become drier still, possibly, as Dr. Burder suggests, partly from the extension of buildings and pavements. On the other hand, the growth of Bristol tends to deteriorate the atmosphere, at least during the prevalence of easterly and south-easterly winds; and our November fogs have certainly been aggravated from this cause.

I must confess to a certain amount of scepticism in dealing with meteorological observations made at watering places. It is notoriously difficult to select absolutely fair situations for one's instruments, and a somewhat dry and warm corner is very apt to be convenient in other respects. At Clifton we have been favourably circumstanced, our local observers, Dr. Burder and the late Dr. G. S. Thomson, being men of known scientific eminence and unbiassed judgment. Of late years additional observations have been made by Messrs. Jupp and Rintoul, at Clifton College.

The mean temperature for 15 years (1853-67) appears to have been 48.7° (about half a degree below that of Greenwich), for seven years (1881-7) 49.1° In winter and spring, as in the West of England generally, the mean temperature is above that of Greenwich, in summer decidedly below, and in autumn less so. Snow frequently falls to the east of the Box Tunnel when there is rain or fair weather at Bath and Bristol; but it is in spring that the advantage is most perceptible, vegetation being much more advanced in the West. The rainfall has been already stated: as to snow, in 9 out of 23 winters the greatest depth of snow was less than an inch, and in 15 it was less than two inches: we have less snow than many places to the south of us. Thunder-storms are certainly rather uncommon; our comparative exemption is probably due to the lie of the Mendips and of the Welsh hills. The mean humidity, which averaged 83.1 in 1853-67, was but 81 in the five years 1883-4-6-7-8. In 1887 and 1888, the only years which I can compare, it was less than that of Bournemouth. The number of rainy days (*i.e.* when 0.01 inch or more fell) averaged, in the five years 1883-7, 170.4 (Burder).

Such being the climate of Clifton, let us proceed to examine its rate of mortality.

In 1875 I read, in the Statistical Section of the British Association, a paper "On the Death-rate of some Health-Resorts, and especially of Clifton." Therein I compared, so far as accessible data allowed me, the death-rates of all such districts in England as resembled Clifton in social character and constitution of population. The figures, taken simply from the Registrar-General's returns for 1871 and 1872, varied from 16.2 for Newton Abbot (Torquay) and 16.3 for Clifton, up to 21.9 for Brighton and 23 for Scarborough. By adding the deaths of persons belonging to the parish, but dying outside its limits in hospitals, workhouses, &c., I arrived at a death-rate of about 16.9, of which the zymotic rate was 1.35. These were very low figures, the former being 4.9 less than should have occurred in a population similarly constituted as to age and sex according to the English Life Table.

Since that time, however, matters have improved further, in Clifton as well as in England generally. The average death-rate in Clifton for the years 1886-87-88 was only 13.3 per thousand, or, after making full allowance for deaths in public institutions, &c., at most 14.6. This is extremely small, especially if we remember that it applies to the entire parish, including the crowded and poor population of the Hotwells as well as the well-to-do community on the upper levels. The zymotic rate averaged only .95, and that from phthisis .88—both remarkably low;—the respiratory death-rate was 2.6. In the adjoining district of Westbury, which geographically and socially resembles the *upper* level of Clifton, the figures were .58, .84, and 1.61. Corresponding rates for all England in 1886 (the latest year for which we have full data) were 2.6, 1.7, and 3.7.*

To sum up, Clifton is a place which combines in an unusual degree the advantages of town and country, Its high standard of healthiness is no doubt due largely to

* These rates should be augmented, perhaps by as much as 10 per cent., for deaths of persons belonging to Clifton or Westbury, but dying in public institutions outside of these parishes. After this addition, and without any reduction for the deaths of extraneous invalids, the figures continue to be remarkably favourable. To be precise, the phthisical mortality of Clifton with Westbury for the three years 1886-7-8 was less than that of any one of the 680 districts of England and Wales during the last decade (1871-80). It may be objected that this comparison is unfair, inasmuch as the mortality from phthisis throughout the whole country has fallen considerably of late years; so that if we could now quote the mortality-rates for 1886-7-8 of all the 680 districts, we should pretty surely find a few which would stand better than Clifton. This is highly probable; but the statistics for the first of these three years, which are fortunately available, enable me to say that in 1886, at least, there was no district in or around the metropolis, no large town (with one curious and perhaps accidental exception), no part of Kent, Sussex, or Devon, and no district containing a known health-resort, or enjoying a special reputation for its climate, which could vie with Clifton as regards comparative exemption from phthisis. That this privilege is due to climatic advantages becomes highly probable when we note that it is shared, more or less, by a large circumjacent region, containing eight counties, and corresponding roughly with the basin of the Severn. In 1871-80, out of 90 registration districts in which the mortality from phthisis was less than 1.5 per 1000, no less than 44 were included in these eight counties. See also, on this point, Haviland's *Geography of Phthisis.*

good sanitary administration and a good (unventilated) system of sewers, but also to some extent to the natural advantages of its site. Its climate is commonly called relaxing, but is not found to be so by strangers from Devonshire, South Wales, or the Severn Valley. Be that as it may, there is plenty of ozone in the atmosphere, except when the wind blows from over Bristol; and if people do not live very fast, they are not in such a hurry to die as in some more bracing places. Changes are frequent rather than great. The prevailing and strongest winds are westerly and south-westerly, with a slightly marine character. East winds are less troublesome than in most parts of England, though at times bitter enough. North-west winds bring thunder, hail, and beautiful skies beloved of artists. The winters as a rule are rather mild, the springs are as fine as anywhere in England, the summers are cool, the late summer and early autumn rainy, the end of autumn foggy. Many invalids might do well by spending the beginning of winter on the Sussex coast, and coming hither, or to some other western station, after Christmas. Asthmatics often do well here, especially those coming from the north and east. Dr. C. J. B. Williams told me that, of all the places to which he had ever sent asthmatics, Clifton and Budleigh Salterton had best deserved the recommendation. And a wealthy old *poitrinaire*, who had spent twenty years in wandering about all the most famous health-resorts, went so far as to tell Dr. Walshe that "the north side of the Old Mall at Clifton was the safest place in Europe." I ought to record my obligations to Dr. David Davies, medical officer of Health for Bristol and Clifton, and to other gentlemen whose names appear in the text, for assistance kindly rendered in the verification of the facts of this paper.

